# A New Method Proposed for Analyzing Airflow Dynamics in Negative Pressure Isolation Chambers Using Particle Image Velocimetry

**DOI:** 10.3390/bioengineering12030302

**Published:** 2025-03-17

**Authors:** Min Jae Oh, Jung Min Moon, Seung Cheol Ko, Min Ji Kim, Ki Sub Sung, Jung Woo Lee, Ju Young Hong, Joon Sang Lee, Yong Hyun Kim

**Affiliations:** 1 AI &Energy Research Center, Korea Photonics Technology Institute, Gwangju 61007, Republic of Korea; hso5922@kopti.re.kr (M.J.O.); mjm7757@kopti.re.kr (J.M.M.); 2 School of Mechanical Engineering, Yonsei University, Seoul 03722, Republic of Korea; kscv0412@gmail.com (S.C.K.); joonlee@yonsei.ac.kr (J.S.L.); 3 Emergency Medicine, Yonsei University College of Medicine, Seoul 03722, Republic of Korea; minzhi@yuhs.ac; 4 SS-ENG Co., Ltd., Bucheon 14449, Republic of Korea; kssung@ss-eng.net (K.S.S.); corono@naver.com (J.W.L.)

**Keywords:** negative pressure isolation chamber, PIV, infection prevention

## Abstract

The COVID-19 pandemic has highlighted the significant infection risks posed by aerosol generating procedures (AGPs). We developed a hood that covers the patient’s respiratory area, incorporating a negative pressure system to contain aerosols. This study analyzed the movement and containment of aerosols within a developed negative pressure isolation chamber. Using particle image velocimetry (PIV) technology, in the optimized design, the characteristics of aerosols were analyzed under both negative and non-negative pressure conditions. The results demonstrated that in the absence of negative pressure, droplets dispersed widely, with diffusion angles ranging from 26.9° to 34.2°, significantly increasing the risk of external leakage. When negative pressure was applied, the diffusion angles narrowed to 20.0–35.1° and inward airflow effectively directed droplets away from the chamber boundary, preventing external dispersion. Additionally, sensor data measuring particle concentrations confirmed that droplets smaller than 10 µm were fully contained under negative pressure, strongly supporting the chamber’s effectiveness. The strong agreement between PIV flow patterns and sensor measurements underscores the reliability of the experimental methodology. These findings highlight the chamber’s ability to suppress external leakage while offering superior flexibility and portability compared to conventional isolation systems, making it ideal for emergency responses, mobile healthcare units, and large-scale infectious disease outbreaks.

## 1. Introduction

The rapid global spread of COVID-19 in 2019 underscored the urgent need for effective infection prevention measures, especially for high-risk infectious diseases. Similarly to COVID-19, the 2003 outbreak of severe acute respiratory syndrome (SARS) demonstrated the potential for transmission even within confined spaces like airplanes, suggesting that airborne droplet transmission can occur through respiratory activities such as coughing and sneezing [1]. COVID-19 primarily spreads via respiratory droplets and aerosols, which can remain airborne for extended periods [2]. Studies show that droplets generated by coughing have a geometric mean diameter of approximately 13.5 µm, while those produced during speaking measure around 16.0 µm [3]. Viruses can be present in small airborne droplets, facilitating infection beyond a distance of 1–2 m from an infected individual. Additionally, the average concentration of respiratory droplets expelled during speech has been reported as 0.04 cm^3^ and 0.16 cm^3^ for droplets with diameters of 3.5 µm and 5 µm, respectively [4]. COVID-19 is highly transmissible and capable of causing severe outcomes through airborne transmission [5]. Before the development of vaccines, blocking all potential transmission routes was essential to mitigate the virus’s spread. Effective infection control measures, including localized exhaust ventilation, high-efficiency air filtration, and ultraviolet disinfection, were critical for preventing airborne transmission and reducing the risk of infection [6]. However, the rapid airborne transmission of COVID-19 has overwhelmed isolation facilities, resulting in delays in both treatment and isolation. The shortage of negative pressure isolation rooms has been associated with patients dying while awaiting care. This highlights the urgent need for alternative methods that can replicate the effects of negative pressure isolation in standard indoor environments to meet emergency isolation requirements for airborne infectious diseases.

Conventional negative pressure isolation rooms face significant challenges in meeting immediate demand during large-scale infectious disease outbreaks due to their high costs and complex infrastructure requirements [7]. The sealed structure of these rooms can lead to the accumulation of internal infectious agents, posing additional risks to both healthcare workers and patients [8]. Additionally, their fixed design limits flexibility in emergency situations and restricts their applicability outside of hospital environments. To address these limitations, this study aims to evaluate the design and effectiveness of a negative pressure isolation chamber as a practical alternative.

Closed isolation tools designed to replace negative pressure isolation rooms include isolation chambers made of transparent acrylic or polycarbonate sheets. These chambers are equipped with suction devices to prevent external emissions and provide additional protection by isolating individuals from external contact [9]. However, such closed systems may trap airborne pathogens inside the chamber, leading to an increased risk of viral accumulation. When opened, the sudden release of stagnant air can result in significant aerosol dispersion, potentially exposing healthcare workers and the surrounding environment to infectious particles [10]. While closed chambers effectively prevent external leakage, they pose an increased risk of infection due to the accumulation of internal infectious agents when opened. Therefore, to minimize the risk of infection, it is essential to design systems that generate negative pressure, control internal airflow, and suppress the external release of infectious agents.

Unlike fully enclosed isolation chambers that cover the entire body, the negative pressure isolation chamber covers the head and part of the chest. This design prevents droplets from leaking outside the chamber through its structural features and suction mechanisms. To validate this design, this study employed particle image velocimetry (PIV) to analyze droplet movement. PIV is an optical flow measurement technique that allows for the precise observation of velocity fields without disrupting the flow itself, making it highly effective for analyzing droplet dynamics [11]. Infectious particles, such as droplets, are typically expelled during sneezing or speaking, and their flow behavior is crucial for understanding viral transmission pathways. Previous research has measured cough velocity profiles and waveforms using simulation devices and analyzed cough velocity fields through PIV [12]. Comparative studies on airflow dynamics during actual coughing and speaking revealed that the initial cough velocity averaged 15.3 m/s for men and 10.6 m/s for women [13]. Additionally, studies comparing flow fields with and without masks demonstrated that while masks do not completely block droplet transmission, they significantly reduce the risk of infection, as analyzed using PIV techniques [14]. These prior studies have contributed to a deeper understanding of droplet flow behavior and confirmed that PIV is a valuable tool for evaluating droplet transmission pathways and assessing infection risks.

Recognizing the need for practical applications of these findings, our research team, which includes board-certified emergency physicians and mechanical engineers, has been developing a novel respiratory barrier enclosure with a negative pressure generator. The purpose of this device is to isolate the patient’s respiratory area during medical procedures. A key feature of this novel enclosure is a double-layered membrane system that seals the patient’s chest area and can easily be converted into a system-wide isolator, resembling a negative pressure isolation chamber. The intubation hood, which covers the patient’s head and neck, has four access orifices for healthcare workers [15,16].

Our team has reported the efficacy of this newly developed respiratory barrier enclosure in containing droplets during procedures such as intubation and CPR. We also assessed the efficiency of procedures like endotracheal intubation through simulation studies. Although procedural times showed no statistically significant difference after 10 min of adequate practice, participants reported discomfort during intubation due to the endotracheal tube frequently touching the ceiling of the hood [15]. While enlarging the hood size could alleviate this discomfort, our team restricted the size of the intubation hood to ensure it remains useful for imaging procedures, including CT scans and coronary angiography. Based on these findings, we concluded that further work is needed to improve both efficacy and efficiency. Additionally, our preliminary studies indicated a lack of established standard methods for accurately testing the barrier effectiveness of such chambers.

Furthermore, in our preliminary studies [15,16], a negative pressure isolation chamber is portable and can be moved with the patient, offering flexibility for various procedural scenarios. These features make a negative pressure isolation chamber a viable alternative to conventional negative pressure isolation rooms or fully enclosed isolation chambers. Its adaptability to individual patient needs and its ability to enhance spatial efficiency address the challenges posed by the shortage of negative pressure isolation rooms.

The objective of this study is to verify that a negative pressure isolation chamber can effectively suppress the external leakage of virus-containing droplets generated by respiratory activities such as sneezing. Unlike conventional closed isolation tools, which may trap airborne pathogens and release them upon opening, the negative pressure isolation chamber actively controls airflow to prevent viral accumulation. This study aims to compare the airflow behavior between negative pressure and non-negative pressure conditions, demonstrating the effectiveness of a negative pressure system in reducing the risk of airborne transmission. To achieve this, PIV technology is employed to visualize and analyze airflow patterns and droplet dispersion, providing quantitative validation of the chamber’s ability to contain aerosolized particles.

## 2. Methods

### 2.1. Design of a Hood

**Figure 1:**
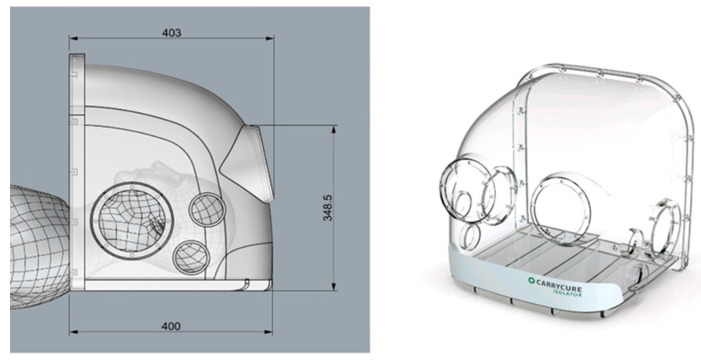
The intubation hood used in the previous study.

The intubation hood was designed with dimensions constrained about 500 mm in width, length, and height, each with four patient access orifices. The intubation hood is intended to cover the patient’s head and neck area, shielding it from the surrounding environment. A similar device, modeled after the chamber shown in Figure 1, was constructed to facilitate experiments and PIV analysis, enabling the measurement of droplet dynamics and flow behavior.

### 2.2. Negative Pressure Generator (NPG)

**Figure 2:**
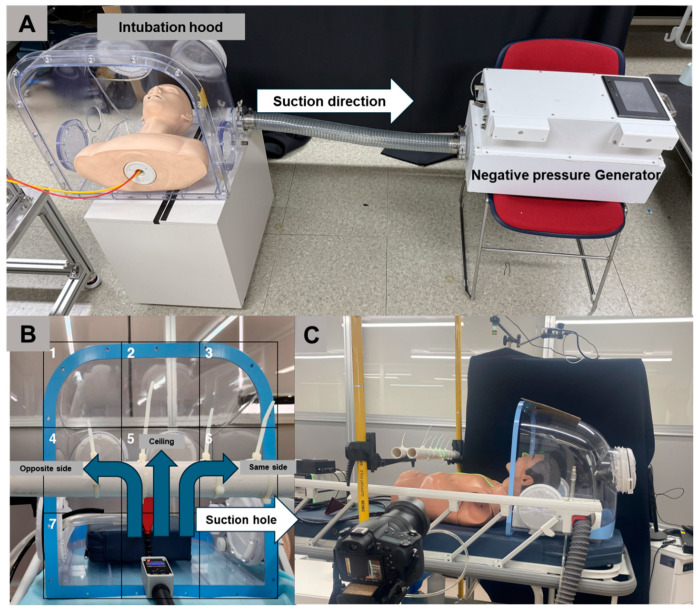
(**A**) An example of a negative pressure generator (NPG) connected to the intubation hood with a patient. (**B**) The open area of the intubation hood was divided into nine sections, with aerosols dispersed in three directions (blue arrows); the suction hole was located on the right lateral inside of the intubation hood (white arrow). (**C**) Particle image velocimetry (PIV) setup illustrating the camera and laser alignment.

A negative pressure generator (NPG) was designed to continuously suction air inside the hood through an 80 mm diameter duct, allowing it to pass through a HEPA filter with 99.7% efficiency in the previous study [17]. Ventilation fans were installed inside the NPG, designed to operate up to four levels of boosters depending on the negative pressure differential inside and outside of the intubation hood. The system was programmed to activate an additional booster if the pressure differential dropped below −10 Pa and to deactivate the booster if the pressure differential exceeded −20 Pa. The pressure differential was continuously monitored using a sensor (differential pressure transmitter ©Beck 93 Sensortechnik GmbH, Steinenbronn, Germany) during suction, as shown in Figure 2A.

Figure 2B shows a frontal view of the NPG setup, indicating the airflow direction toward the suction duct. This visualization highlights the uniform suction effect across the hood. Figure 2C illustrates the PIV setup used to analyze droplet dynamics and airflow behavior, providing a detailed representation of the laser plane and camera alignment used in the experiment.

Without the double-layered membrane, the pressure differential between the inside and outside was maintained below −10 Pa, ensuring that all four boosters remained active. When the hood was completely closed, the pressure differential exceeded −80 Pa with all four boosters running. In this study, the hood was kept in an open state, and its barrier efficiency was assessed under conditions with all four boosters running automatically.

### 2.3. Experimental Setup for PIV Analysis

**Figure 3:**
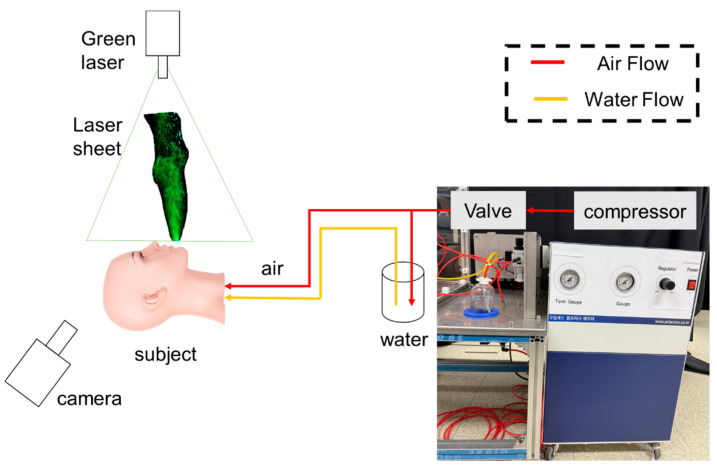
Configuration of the aerosol-generating simulation device.

In this study, a simulated aerosol-generating device was used to replicate the conditions of a patient’s sneeze, and the flow characteristics of droplets were analyzed using PIV techniques. The setup, as shown in Figure 3, included an air compressor to generate a mixture of water and air for droplet expulsion, a green laser for PIV analysis, and a Sony RX10M4 camera (Sony, Tokyo, Japan) to capture droplet movement.

The device was designed to mimic a patient sneezing while lying down with a horizontally positioned mannequin and a laser sheet aligned perpendicularly to the droplet outlet for a precise observation of flow dynamics.

Sneeze velocities typically range from 15 to 30 m/s [9]. The experimental setup was designed to replicate these sneeze-like conditions, using a 2D laser plane in a darkroom to enhance droplet visualization. Compressed air mixed with water droplets was expelled through a spray device, with an initial pressure of approximately 0.05 MPa and a compression duration of 0.9 s.

**Figure 4:**
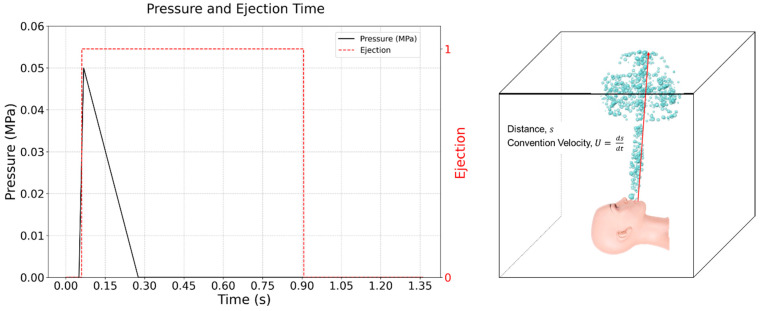
Convection velocity calculation based on pressure and ejection.

As shown in Figure 4, the vertical distance *s* from the ejection outlet to the ceiling was measured to be 0.45 m. The aerosol velocities were measured by particle image velocimetry, where the aerosol generation process is visualized as a plume of smoke being expelled. The vertical distance from the end of the plume to the aerosol initiation point is defined as distance, and the convection velocity is defined as the temporal derivative of *s* (U = ∆s/∆t). Using these principles, the convection velocity under the experimental conditions was calculated to range between 15 and 20 m/s.

The air compressor, equipped with an adjustable air control valve, allowed for the regulation of air discharge intensity and duration, ensuring realistic high-speed ejection parameters. These were crucial for replicating sneeze dynamics and assessing droplet leakage in the negative pressure isolation chamber.

To accurately analyze the flow characteristics of droplets using PIV experiments, key parameters such as laser wavelength, laser power, and camera shutter speed were optimized, as shown in Table 1. A green laser (530 nm) was used to optimize droplet visibility. This wavelength enhances the clarity of fine particles like droplets, making them distinctly visible. Moreover, it optimizes the signal-to-noise ratio (SNR) when captured with a high-speed camera, enabling a precise analysis of droplet flow patterns. The laser power was set to 135 mW to ensure that the droplets appeared sufficiently bright, with sharp contours for clear visualization.

Camera settings were meticulously adjusted to capture the high-speed motion of droplets with clarity in a darkroom environment. The shutter speed was set to 1/500 s to eliminate motion blur and accurately capture fast-moving droplets. The aperture was set to f/3.5 to allow adequate light into the camera, while the ISO value was set to 4000 to provide sufficient brightness in the darkroom while minimizing image noise caused by excessively high ISO levels. These well-optimized settings ensured that the PIV analysis could accurately capture and analyze the dynamics of droplet flow during the simulated sneeze experiments.

### 2.4. Image Preprocessing

**Figure 5:**
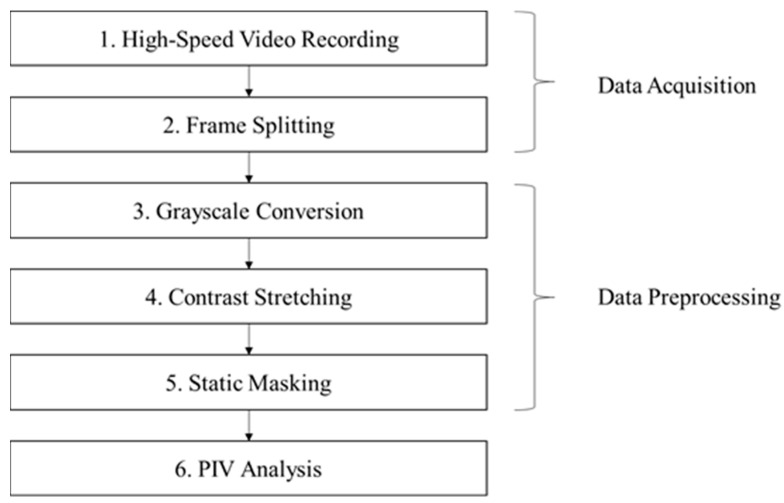
Flowchart for PIV analysis.

To enhance the accuracy of PIV analysis, image preprocessing was performed to improve the visibility of droplet flow during aerosol generation. Key steps included converting RGB images to grayscale, applying contrast stretching, and using static masking to isolate relevant regions of interest (ROIs). Figure 5 provides an overview of the preprocessing steps, showing the workflow from raw image data to the final processed output.

RGB images were converted to grayscale to streamline data processing and enhance droplet visibility. The grayscale value for each pixel was calculated as

(1) Gray=0.299×R+0.587×G+0.114×B

This conversion reduced the complexity of the image and emphasized brightness differences, making droplets more visually distinct.

To further clarify droplet contours, contrast stretching was applied to expand the pixel value range. This process adjusted brightness differences using the following formula:

(2) $$p^{\prime }=\frac{p-p_{min}}{p_{max}-p_{min}}\times \left(d_{max}-d_{min}\right)+d_{min}$$

- p: pixel value;
- $p_{min}$: minimum pixel value in the image;
- $p_{max}\;:$ maximum pixel value in the image;
- $d_{min}\;:$ minimum adjusted pixel value (0);
- $d_{max}\;:$ maximum adjusted pixel value (255).

$p_{min}$ and $p_{max}$ represent the darkest and brightest pixel values, respectively, and $d_{min}$ and $d_{max}$ define the adjusted pixel range (0 to 255).

Static masking was employed to remove unnecessary background regions, isolating the areas where droplets were expelled. This ensured that irrelevant elements did not interfere with the analysis. The masking operation was defined as

(3) $$mask(x,y)=\{\begin{array}{cc} 0, & if\;(x,\;y)\;is\;background\;region\\ I(x,y) & if(x,y)\;is\;ROI(Region\;of\;Interest) \end{array}$$

**Figure 6:**
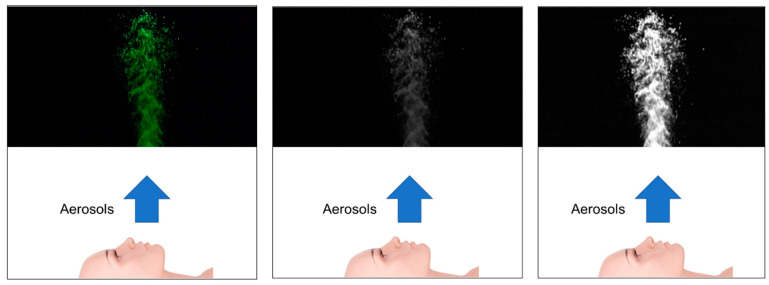
From left to right: original, grayscale, contrast stretching.

The final results after applying static masking are shown in Figure 6, where unnecessary background regions are removed and the droplet region of interest is isolated for analysis.

### 2.5. Statical Analysis

The statistical approach employed in this study, including the Shapiro–Wilk test for normality assessment, Welch’s *t*-test for comparing group differences, and Cohen’s d for effect size estimation, aligns with methodologies described in previous research, ensuring robust statistical inference and valid comparisons between conditions [18].

The study aimed to evaluate the effectiveness of the negative pressure isolation chamber in controlling aerosol dispersion by comparing dispersion angles under negative pressure and non-negative pressure conditions. Statistical analysis often assumes that data follow a normal distribution. To ensure the appropriateness of parametric tests, the normality of the data was first assessed using the Shapiro–Wilk test. The test statistic is given by

(4) $$W=\frac{{\left(\sum_{i=1}^{n}a_{i}X_{\left(i\right)}\right)}^{2}}{\sum_{i=1}^{n}{\left(X_{i}-\bar{X}\right)}^{2}}$$

where *W* is the test statistic, $X_{\left(i\right)}$ represents ordered sample values, and $a_{\left(i\right)}$ are constants derived from expected normal values. A *p*-value < 0.05 indicates that the data significantly deviate from normality.

To compare the dispersion angles between the two conditions, a Welch’s *t*-test was applied. Welch’s *t*-test is preferred over a standard two-sample *t*-test, as it accounts for possible unequal variances between groups. The test statistic is calculated as

(5) $$t=\frac{{\bar{X}}_{1}-{\bar{X}}_{2}}{\sqrt{\frac{s_{1}^{2}}{n_{1}}+\frac{s_{2}^{2}}{n_{2}}}}$$

where ${\bar{X}}_{1}$ and ${\bar{X}}_{2}$ are the sample means, $s_{1}^{2}$ and $s_{2}^{2}$ are the sample variances, and $n_{1}$ and $n_{2}$ are the sample sizes of each group. Welch’s *t*-test adjusts for unequal variances using the following degrees of freedom (*df*):

(6) $$df=\frac{{\left(\frac{s_{1}^{2}}{n_{1}}+\frac{s_{2}^{2}}{n_{2}}\right)}^{2}}{\frac{{\left(\frac{s_{1}^{2}}{n_{1}}\right)}^{2}}{n_{1}-1}+\frac{{\left(\frac{s_{2}^{2}}{n_{2}}\right)}^{2}}{n_{2}-1}}$$

If *p* < 0.05, the difference between the two groups is considered statistically significant.

To quantify the magnitude of the observed differences, Cohen’s d was computed as an effect size measure. The formula for Cohen’s d is

(7) $$d=\frac{{\bar{X}}_{1}-{\bar{X}}_{2}}{s_{p}}$$

where $s_{p}$ is the pooled standard deviation, given by

(8) $$s_{p}=\sqrt{\frac{\left(n_{1}-1\right)s_{1}^{2}+\left(n_{2}-1\right)s_{2}^{2}}{n_{1}+n_{2}-2}}$$

According to established guidelines, a value above 0.8 is considered a large effect size, indicating a substantial difference between the two conditions.

A significance level of α = 0.05 was applied to all statistical tests, meaning that there is a 5% probability of committing a Type I error—incorrectly rejecting the null hypothesis when it is true. This threshold is widely used in scientific research to balance Type I and Type II errors, ensuring a reasonable trade-off between false positives and false negatives in hypothesis testing.

## 3. Results

This study aimed to verify the effectiveness of the negative pressure isolation chamber in preventing external droplet leakage by analyzing their flow characteristics within the chamber. The chamber’s negative pressure was set to 2.0 Pa with an airflow velocity of 0.5 m/s at the opening. According to the Centers for Disease Control and Prevention (CDC), negative pressure isolation rooms are recommended to maintain a minimum pressure of 2.5 Pa and an airflow velocity of 0.2–0.5 m/s [19]. Although the pressure used in this study was slightly below the CDC recommendation, the airflow velocity met the guidelines, ensuring the effective containment of contaminants.

**Figure 7:**
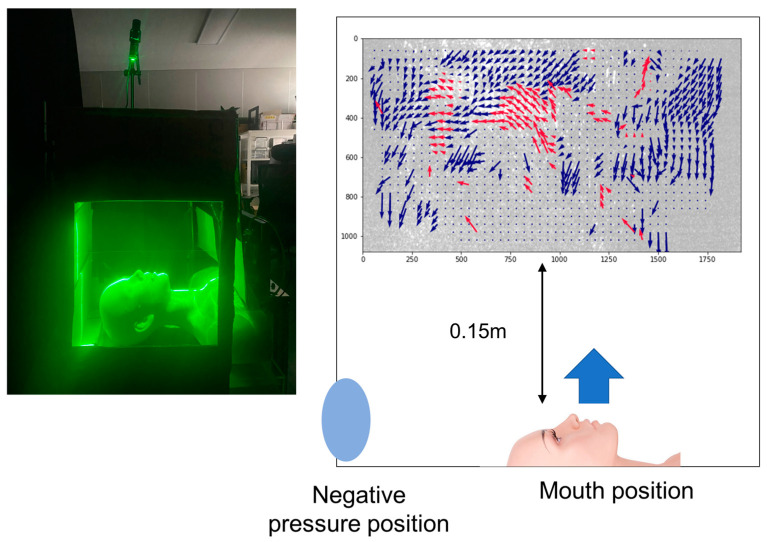
Examples of the negative pressure isolation chamber and PIV analysis, where red arrows in the PIV result indicate upward airflow and blue arrows represent downward airflow.

The negative pressure source was positioned on the left side of the chamber to allow healthcare workers to perform procedures without obstruction. This configuration maintained a clear working area while effectively preventing the external leakage of infectious agents. Figure 7 provides an overview of the experimental setup and PIV analysis results. On the left, a mannequin simulates aerosol generation inside the chamber. The right side of Figure 7 shows the PIV analysis results 1.5 s after aerosol generation.

To accurately analyze droplet flow, the PIV measurements were taken at a distance of 0.15 m above the mouth. This distance was chosen because droplets immediately expelled from the mouth are highly concentrated and have strong velocities, making PIV analysis challenging due to the high particle density and rapid motion. By measuring at this distance, the analysis provided a clearer visualization of the airflow patterns and droplet behavior.

### 3.1. Analysis of Droplet Flow Inside the Negative Pressure Isolation Chamber

**Figure 8:**
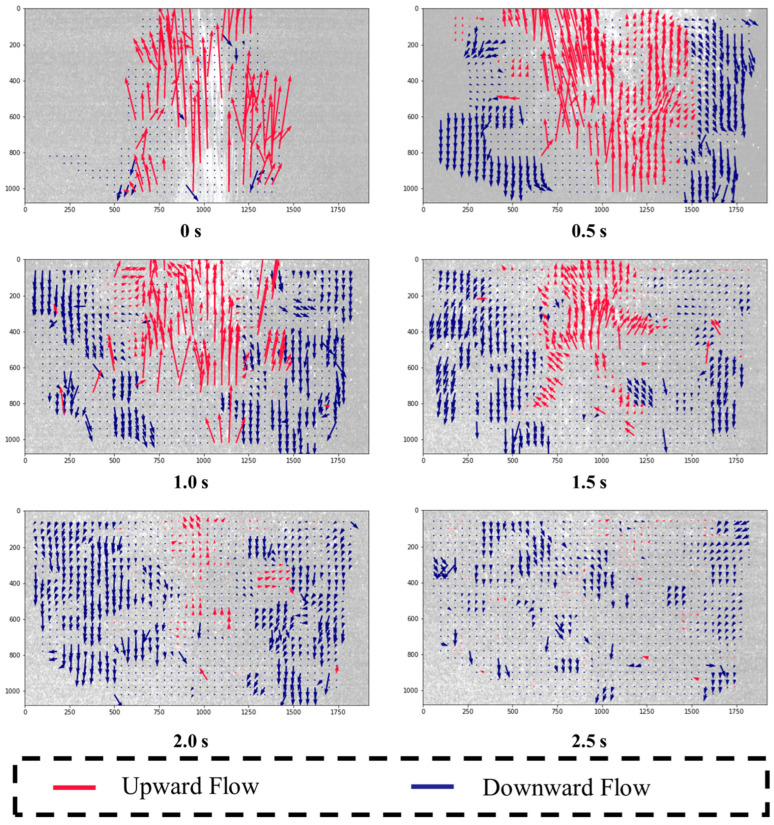
Droplet flow inside the negative pressure isolation chamber (without negative pressure).

The containment effectiveness of the chamber was evaluated by comparing droplet flow patterns before and after applying negative pressure. Without negative pressure, droplets primarily traveled in the sneeze’s direction, with some adhering to or reflecting off the upper surface, as shown in Figure 8. Droplets along the chamber boundaries indicated a potential risk of leakage.

PIV analysis revealed that after aerosol generation, droplets reflected from the upper surface descended and were drawn back toward the center by vortices created by the main airflow. This behavior is consistent with droplet flow characteristics observed after coughing [20]. Larger droplets settled quickly, while smaller droplets, influenced by airflow, moved toward the outlet.

**Figure 9:**
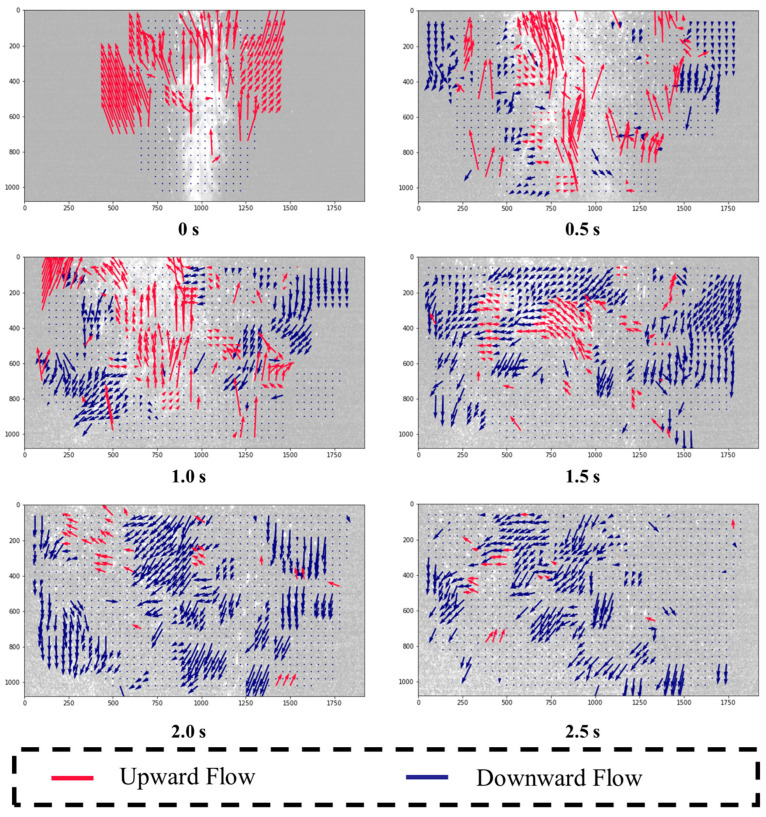
Droplet flow inside the negative pressure isolation chamber (with negative pressure).

When negative pressure was applied, as illustrated in Figure 9, droplets were actually directed inward, preventing external leakage. The pressure difference created by the negative pressure system ensured that droplets remained contained within the chamber, suppressing their dispersion outside.

### 3.2. Aerosol Airflow Boundary and Angle Analysis

This experiment aimed to analyze the airflow boundary and diffusion angles caused by coughing or sneezing inside the negative pressure isolation chamber, with the patient in a supine posture. Using PIV, velocity distributions in both horizontal and vertical directions were measured and normalized between −1 and 1. The airflow boundary, where velocity transitions from positive to negative, distinguished upward (red) and downward (blue) flow regions through derived streamlines. The horizontal velocity distribution reflected upward spreading during aerosol generation, while the vertical distribution showed boundary changes.

**Figure 10:**
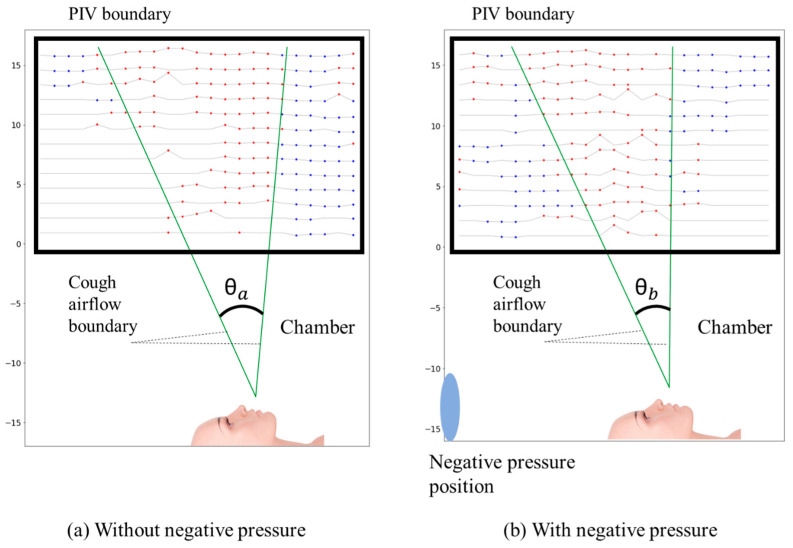
Comparison of airflow boundaries and diffusion angles under non-negative and negative pressure conditions, with boundary angles determined by the separation between upward airflow indicated by red dots and downward airflow indicated by blue dots.

**Figure 11:**
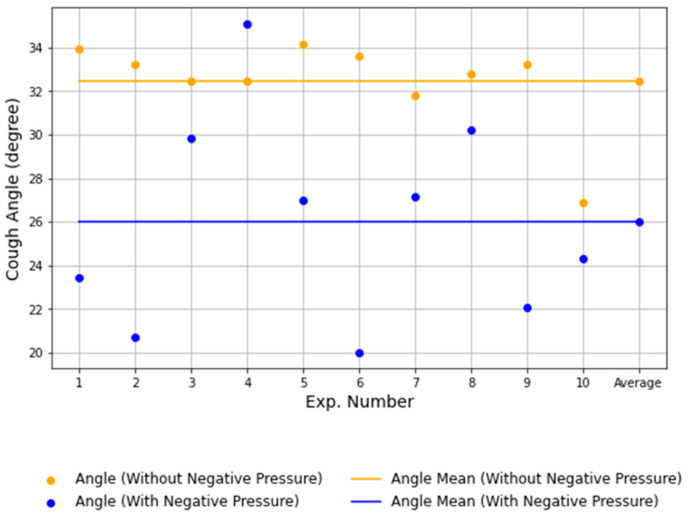
Comparison of diffusion angle ranges and averages under non-negative and negative pressure conditions.

Figure 10 and Figure 11 illustrate the differences in airflow diffusion angles under non-negative and negative pressure conditions. In the non-negative pressure condition, the diffusion angle $\theta_{\alpha }$ ranged from 26.9° to 34.2° with a mean of 32.5°, spreading widely with greater variation. Conversely, under negative pressure, the angle $\theta_{b}$ narrowed to a range of 20.0° to 35.1° with a mean of 26.0°. The airflow boundary bending inward and the variation significantly reduced. Figure 11 visually confirms these statistical findings, showing that the non-negative condition has a broader angle distribution, while the negative pressure condition exhibits more focused and converged airflow.

To further evaluate the statistical significance of these differences, a Shapiro–Wilk test was conducted to assess normality. The results, calculated according to Equation (4), indicated that the negative pressure condition followed a normal distribution (*p* = 0.6909), whereas the non-negative pressure condition did not (*p* = 0.000802). Since the normality assumption was violated for the non-negative pressure condition, Welch’s *t*-test was used to compare the dispersion angles between conditions, as it accounts for unequal variances.

The t-value, calculated using Equation (5), was t = 3.9106. Since Welch’s *t*-test adjusts for unequal variances, the degrees of freedom were calculated using Equation (6), yielding *df* = 16.82. Given that *p* = 0.001974 was below α = 0.05, the result was statistically significant, confirming the negative pressure chamber’s effectiveness in controlling aerosol spread.

The dispersion angle mean (*M*) and standard deviation (*SD*) were lower in the negative pressure condition (*M* = 26.0°, *SD* = 4.1°) than in the non-negative pressure condition (*M* = 32.5°, *SD* = 3.2°), indicating reduced variability and improved airflow containment.

The effect size, calculated using Equation (7) with the pooled standard deviation from Equation (8), was d = 1.75. As this exceeds the 0.8 threshold, the observed difference is classified as large, indicating a substantial impact of the negative pressure condition.

These statistical findings, supported by Figure 10 and Figure 11, confirm that the negative pressure condition effectively reduces aerosol dispersion and improves airflow containment. These results suggest that implementing negative pressure environments can be an effective strategy for minimizing airborne contamination in various applications, including healthcare settings and laboratory environments.

### 3.3. Droplet Concentration Measurement at the Chamber Boundary

While the PIV analysis in Section 3.3 demonstrated changes in airflow patterns and diffusion angles, further validation was conducted to measure actual droplet leakage. To quantify droplet dispersion, a particle concentration sensor was installed at the chamber’s open boundary to monitor droplets smaller than 10 µm.

Droplets smaller than 10 µm were specifically measured because these particles dominate aerosols produced during aerosol generation. Studies indicate that the majority of droplets generated by patients with upper respiratory tract infections fall within the size range of 0.5 to 10 µm, accounting for over 85% of the total particle count [21]. These small droplets, classified as aerosols, remain airborne for extended periods due to their low settling velocity, allowing them to travel significant distances. Moreover, these aerosols play a critical role in disease transmission as they can penetrate deep into the respiratory tract upon inhalation and often carry higher viral loads compared to larger droplets. Thus, monitoring droplets smaller than 10 µm is essential for accurately evaluating the containment efficiency of the intubation hood in preventing aerosol leakage.

According to [22], larger droplets (≥50 µm) rapidly settle due to gravitational forces, whereas smaller droplets (≤10 µm) remain suspended in the airflow and disperse over greater distances. Based on this principle, pixel intensity was used to estimate droplet concentration in regions excluding the main airflow stream. The grayscale intensity of each pixel was correlated with droplet size, where higher intensity values indicated a higher presence of small droplets (≤10 µm). This relationship was validated by comparing pixel intensity histograms with particle sensor measurements, confirming that 90.51% of detected droplets were smaller than 10 µm. This validation ensured that PIV-based droplet visualization accurately reflected actual aerosol dispersion patterns.

**Figure 12:**
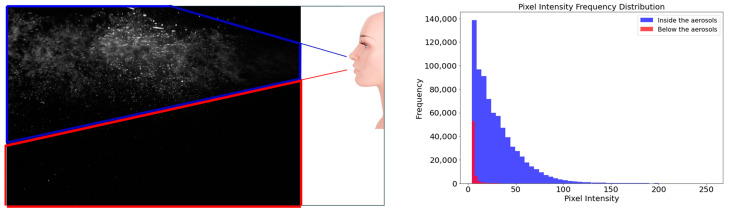
Visualization of horizontally dispersed aerosols from a simulated cough and pixel intensity distribution for particle size verification.

Figure 12 illustrates these findings. The left image visually represents the aerosol dispersion, with the blue box highlighting aerosols predominantly containing droplets smaller than 10 µm following the turbulent flow. The red box marks the region where larger droplets (≥10 µm) have settled due to gravity. The right graph quantifies these observations, showing pixel intensity distributions for each region. Blue bars correspond to aerosols in the blue box, and red bars represent particles in the settled region marked by the red box.

The analysis of pixel intensity distributions revealed that 90.51% of the particles were smaller than 10 µm, confirming the dominance of particles under 10 µm in the aerosols.

**Figure 13:**
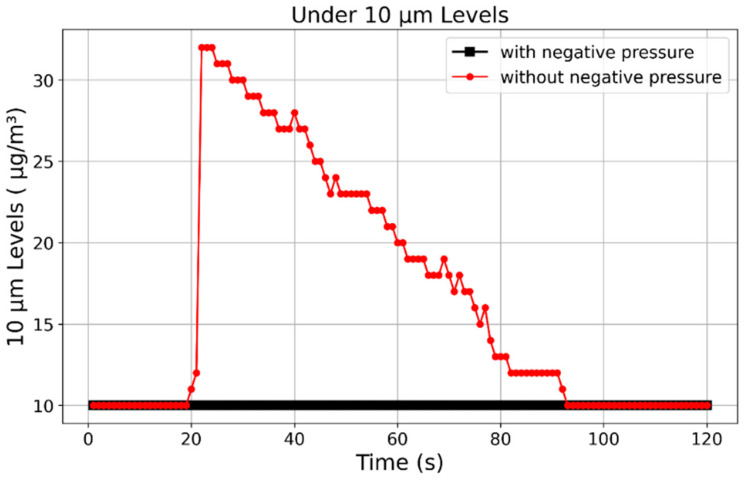
Comparison of droplet leakage inside the negative pressure isolation chamber.

Figure 13 compares droplet leakage under different pressure conditions. Without negative pressure, a sharp increase in droplet concentration was observed, confirming significant leakage. In contrast, under negative pressure, no substantial changes in droplet concentration were detected, validating the system’s ability to effectively contain aerosols and minimize the risk of external dispersion.

Overall, the experimental results demonstrated that the negative pressure isolation chamber effectively controlled airflow direction, reduced droplet diffusion angles, and prevented external leakage, confirming its potential as a reliable containment system for infection control.

## 4. Discussion

This study confirms the effectiveness of the negative pressure isolation chamber in high-risk environments. The reduction in diffusion angles $\theta_{\alpha }$: 26.9–34.2° to $\theta_{b}$: 20.0–35.1°) observed in PIV analysis highlights the critical role of negative pressure in controlling airflow directionality, mitigating respiratory droplet spread, and minimizing leakage during aerosol generation. The results from our PIV analysis confirm that negative pressure isolation chambers effectively regulate airflow, reducing the outward dispersion of respiratory aerosols. In contrast, closed isolation tools without active air circulation may allow airborne particles to remain suspended for longer, potentially increasing the risk of transmission upon chamber opening. This highlights the importance of integrating negative pressure mechanisms into protective isolation devices.

The agreement between PIV flow patterns and sensor measurements validates the experimental setup, highlighting the value of combining visualization techniques with quantitative sensor data to evaluate droplet containment strategies. Unlike fully enclosed isolation systems, the negative pressure isolation chamber provides greater flexibility and portability, making it ideal for various healthcare scenarios, including emergency responses and procedures outside standard facilities.

As this study was conducted under controlled laboratory conditions, future research should test the chamber in dynamic environments with varying airflow, humidity, and droplet compositions to better replicate real-world clinical settings. While negative pressure isolation chambers effectively control airflow and minimize aerosol dispersion, their impact on patient respiratory comfort should be considered. Strong airflow within the chamber may cause respiratory dryness, mucosal irritation, and temperature fluctuations, potentially affecting breathing comfort. Additionally, the altered airflow may require additional respiratory effort, particularly for individuals with pre-existing respiratory conditions.

In contrast to non-negative pressure environments, which rely on natural airflow exchange, negative pressure isolation chambers continuously extract air, potentially increasing respiratory burden if airflow velocity is too high. While this design effectively prevents external contamination, it may also impose additional respiratory effort due to continuous air extraction. This trade-off should be carefully evaluated to balance infection control and patient comfort in long-term applications.

Future studies should evaluate these physiological effects to optimize the design of negative pressure devices for prolonged use. In addition to assessing airflow and aerosol dispersion, expanding research to include different respiratory activities, such as coughing or speaking, would provide a more comprehensive assessment of the chamber’s efficacy.

These findings underscore the negative pressure isolation chamber’s potential as a cost-effective and adaptable alternative to conventional isolation rooms, particularly during large-scale outbreaks where healthcare resources are constrained.

In a separate study, our research team evaluated the effectiveness of a modified intubation hood equipped with negative pressure using the real-time measurement of droplet leakage with polyalphaolefin (PAO) particles. During the experiment, PAO particles were generated inside the hood, and their leakage was measured using an aerosol photometer. Real-time measurements under controlled conditions confirmed that droplet leakage was effectively suppressed to under 0.3%, demonstrating a highly effective containment of aerosol dispersion during medical procedures [14]. Additionally, in our study, we tested droplet leakage using various coughing angles and particle sensors, confirming that there was no droplet leakage. These results align with those of the attached study, further validating the effectiveness of the modified design.

## 5. Conclusions

This study introduced and validated a newly developed negative pressure isolation chamber, designed as a solution for infection prevention. Unlike conventional systems, the open-type design provides flexibility and adaptability without compromising containment performance. The experimental results demonstrated that the chamber effectively prevents the leakage of infectious agents, ensuring safe containment even in high-risk scenarios.

PIV analysis revealed significant differences in airflow behavior within the chamber under negative and non-negative pressure conditions. Without negative pressure, droplets dispersed freely, spreading widely in the horizontal direction. The diffusion angle ($\theta_{\alpha }$) ranged from 26.9° to 34.2° with an average of 32.5°, and a strong downward flow was concentrated along the chamber’s boundaries, increasing the risk of external leakage. In contrast, under negative pressure, the airflow boundary bent inward, directing droplets along a controlled path. The diffusion angle ($\theta_{b}$) narrowed to 20.0–35.1°, with an average of 26.0°. These results demonstrated the chamber’s ability to converge airflow patterns, suppress droplet dispersion, and significantly reduce leakage risks.

Figure 8 and Figure 9 further demonstrated that without negative pressure, droplets adhered to or reflected off the chamber’s upper surface, increasing the risk of leakage. Under negative pressure, however, droplets were effectively directed inward and were contained within the chamber’s boundaries, underscoring its robustness in high-risk scenarios.

Furthermore, the chamber’s modular design facilitates integration with existing medical equipment, such as ventilators and endoscopic devices, allowing for seamless adaptation across a wide range of medical procedures. This adaptability makes it an invaluable tool in both clinical and field settings, especially in managing infectious diseases with high transmissibility.

Additionally, sensor measurements confirmed that droplets smaller than 10 µm were fully contained under negative pressure, further reinforcing the chamber’s reliability in controlling high-risk airborne particles. The strong agreement between PIV flow patterns and sensor measurements underscores the robustness of the experimental setup and methodology.

These findings clearly demonstrate the negative pressure isolation chamber’s ability to prevent leakage, providing robust evidence of its effectiveness in controlling respiratory droplets. Its design ensures high containment efficiency while offering advantages in flexibility and portability. This makes the chamber ideal for diverse healthcare scenarios, including emergency medical responses and procedures conducted outside conventional facilities. Moreover, its scalable and cost-effective design addresses critical challenges in resource-limited settings, particularly during large-scale outbreaks or pandemics.

We plan to use this negative pressure isolation chamber in real-world clinical settings. The system will be implemented in controlled healthcare environments to further evaluate its effectiveness in managing airborne transmission. Future studies will focus on optimizing its performance based on practical use cases and user feedback, ensuring its applicability in diverse medical scenarios.

## Figures and Tables

**Table 1:** Data settings for PIV.

	System Setting Parameter	Value
Laser Setting	Laser power	135 mW
	Laser wavelength	λ = 530 nm
Camera Setting	Image resolution	1920 × 1080 pixel
	Shutter speed	1/500 s
	Aperture	f/3.5
	ISO setting	ISO4000
	High-speed FPS	480 fps (×20 slow)
	Frame interval	0.002 s

## Data Availability

Data are contained within the article.
